# Effect of a feed additive based on a mix of condense and hydrolysable tannins (ByPro^®^) on lactating dairy cows milk production performance

**DOI:** 10.1007/s11250-025-04734-8

**Published:** 2025-11-11

**Authors:** Alejandro R. Castillo, Martin Pol, Mónica Gaggiotti, Julio A. Di Rienzo, Damiano Cavallini

**Affiliations:** 1 University of California, Cooperative Extension, Merced, CA 95340 USA; 2 Lactodiagnóstico Sur SRL, Buenos Aires, 1636 Argentina; 3 Estación Experimental Rafaela, INTA, Rafaela, Santa Fe. Argentina 2300 Argentina; 4 Facultad Cs. Agropecuarias, Universidad Nacional de Córdoba, Córdoba, 5000 Argentina; 5 Dipartimento di Scienze Mediche Veterinarie, Alma Mater Studiorum University of Bologna, Bologna, Italy

**Keywords:** On-farm research, Dairy cows, Condensed and hydrolysable tannins, Lactation performance

## Abstract

The objective of this research was to evaluate the effect of a commercial mix of condensed and hydrolysable tannins feed additive to lactating dairy cows. Two high-scale dairy farm units, similar management and diets were used. All lactating cows in one unit were fed with the mix of tannins (ByPro^®^ 0.3%DMI), the other unit was the Control. Cows were categorized per DIM, 0 to 30, 31 to 120, 121 to 365, and more. Milk yield and composition was evaluated in all the lactating cows in each dairy unit at time 0, (initial), and after 4 weeks of supplementation (final). A linear model was adjusted for each variable. Estimated DMI and diets were similar in both dairy units, averaging 25.5 kg DMI/cow, as well as feed ingredients, dietary nutrient contents, and milk yield (34 kg milk/cow per day). Milk yield, from 0 to 365 DIM was increased (*P <* 0.01) on supplemented cows (+ 2.3 ± 0.33 L milk/cow.d), with no effect on milk fat and protein contents. High data variability was observed on cows with 0–30 DIM, milk fat and protein content were reduced by ByPro without effect on milk yield. Positive responses of ByPro^®^ on milk, milk fat and protein contents from 31 to 120 and 121–365 DIM were observed, + 3.6 and + 3.2 kg milk/cow.d; +0.27% fat and + 0.21% protein; and + 0.17% fat, + 0.11% protein; respectively. The mix of tannins increased milk lactation performance on cows with more than 30 DIM.

## Introduction

The dairy cattle industry plays a crucial role in global food production, providing essential nutrition through milk and dairy products. However, the sector is confronted with a range of challenges that undermine its sustainability and efficiency (Palmonari et al. 2021; Magro et al. 2024). These issues include environmental concerns, such as greenhouse gas emissions and land use, economic pressures driven by fluctuating milk prices and rising feed costs (Gasparini et al. 2024; Koakoski et al. 2024), climate change (Felini et al. 2024; Mortazavi et al. 2025), and ongoing debates about animal welfare in the context of intensive farming practices (Mammi et al. 2021; Cavallini et al. 2025a) and precision livestock farming (Lamanna et al. 2025a; Giannone et al. 2025). This paper explores nutritional solutions designed to enhance system efficiency, reduce environmental impact, and improve animal welfare, aiming to promote a more sustainable and ethical future for dairy production.

Over-feeding proteins in dairy cows may reduce production efficiency (NASEM 2021; Cavallini et al. 2021, 2025b) and increase ammonia emissions from dairy manure and environmental concerns. Excess proteins in lactating dairy diets is excreted mostly as urinary nitrogen (N) (Castillo et al. 2000; Ferlizza et al. 2020).

Nitrogen (N) utilization efficiency in lactating dairy cows is notably low, averaging only 25–28%, with the majority of dietary N being irreversibly lost through mainly urine (Castillo et al. 2000; Zhao et al. 2019). This inefficiency presents significant economic challenges and contributes to environmental concerns, such as ammonia emissions and nutrient pollution (VandeHaar and St-Pierre 2006). Ammonia is an important atmospheric pollutant that plays a key role in several air pollution problems. When combined with nitric acid and NO_x_, ammonia forms aerosol nitrate, which contributes significantly to total particulate matter, PM_2.5_ (McNaughton and Vet 1996).

Therefore, developing feed additives that improve the retention and utilization of dietary nitrogen while minimizing nitrogen losses through excretion is crucial to ensuring the economic sustainability and environmental viability of the dairy industry (Jalal et al. 2025; Rashid et al. 2025).

Tannins are commonly defined as water-soluble polyphenolic compounds ranging in a molecular weight from 500 to 3000 Daltons (Bate-Smith and Swain, 1962). Polyphenolic compounds may occur in plant leaves, roots, trunks, barks, buds, fruits, flowers, and seeds; and it is estimated to be the fourth most abundant biochemical produced by vascular plant tissues after cellulose, hemicellulose, and lignin. Traditionally, they were considered as a plant defensive evolution mechanism of different pathogens and herbivore organisms in general. Because tannins are complex and energetically costly molecules to synthesize, their widespread occurrence and abundance on our planet suggest that tannins play an important role in plants’ function and evolution. Not only as defensive mechanism of vascular plant tissues, also, affecting nutrient cycling in nature by hindering decomposition rates, complexing proteins, inducing toxicity to microbial populations and inhibiting enzyme activities (Kraus et al. 2003; and Muller-Harvey 2006).

Muller-Harvey (2006) reviewed the effects of different types of tannins on animal nutrition and health. Results indicates that tannins form a highly diverse group of natural products with promising nutritional, veterinary, and environmental effects. In the last years, ruminant nutrition research focuses on the ability of tannins to improve nutrient utilization efficiencies (particularly N), animal performance, and to reduce nutrient losses to the environment.

Studies with high milk production lactating dairy cows, feeding a mix of condensed and hydrolyzable tannin extracts improved the efficiency of N utilization, increasing true protein content in milk, with a trend to reduce milk urea nitrogen (MUN), and urinary N excretion (Aguerre et al. 2015, 2016). At the same study, slurry from cows fed the mix tannins were applied to soil-containing lab-scale chambers. Ammonia emissions in slurry were both significantly reduced when cows were fed different levels of proteins and a mix of tannins, or when tannins were directly applied to simulated barn floors (Powell et al. 2011a, b).

Supplementation with tannin extracts (TE) has been reported to confer a range of beneficial effects, including astringency, anti-inflammatory, and antioxidant properties (Sharma et al. 2021). These effects are particularly relevant for early-lactating cows, which are prone to oxidative stress and exhibit reduced antioxidant defenses (Sharma et al. 2011). The antioxidant properties of TE may help mitigate oxidative stress in these animals (Liu et al. 2013). Additionally, TE has been shown to shift nitrogen excretion from urine to feces in dairy cows, potentially reducing environmental pollution (Aguerre et al. 2016). TE supplementation also influences rumen fermentation by partially regulating the rumen microbiota (Grazziotin et al. 2020). However, the effects of TE appear to be dose dependent. While low doses (e.g., 0.45% of dietary dry matter) have been associated with increased milk true protein content, higher doses (e.g., 1.8% of dietary dry matter) have been linked to reduced feed intake, milk yield, and nutrient digestibility (Aguerre et al. 2016, 2020). Based on these findings, we hypothesize that supplementing dairy cow diets with a low dose of quebracho-chestnut tannin extracts could enhance lactation performance and reduce nitrogen excretion by improving rumen fermentation patterns and antioxidant status compared to a control diet.

While previous research on tannin extracts was often conducted in controlled settings with low-variability herds, a significant knowledge gap exists. No large-scale, on-farm studies have evaluated the effects of a commercial tannin mix on the high-variability cow populations managed under typical commercial dairy conditions, particularly across different lactation stages. The objective of this on-farm research was to evaluate the effect of a commercial mix of condensed and hydrolyzable tannins feed additive (ByPro^®^) to lactating dairy cows on milk production performance, milk yield and milk composition.

## Materials and methods

The trial was carried out on a high-scale dairy farm in Argentina. The dairy farm has two split dairy units (free stalls) and about 3000 milking Holstein cows in each unit. Both units have Holstein dairy cows, and similar management practices and diets. All lactating cows in one dairy unit received a feed additive based on a mix of tannins ByPro^®^ and the other dairy unit was used as Control, as reported in previous studies (Castillo et al. 2025). The cows in both dairy units received the same dietary ingredients. The total mixed rations (TMR) diets were based on 7% Alfalfa hay, 26.4% Corn silage, 5% Wheat silage, 4% Alfalfa silage, 5.8% High moisture corn, 14.2% Ground dry corn grain, 20% Corn gluten feed, 7.2% Soybean meal, 5.8% Soybean grain, 1.1% Bypass fat, and 3.5% Mineral and Vitamin Premix. The dietary difference between Control and Treatment group was the mix of tannins included in the TMR.

The dose of the mix-tannins feed additive was 0.3% of the total estimated DMI per cow. The DMI was estimated based on the total daily mixed ration supplied divided by the number of lactating cows and adjusted for DM contents. The DM content in the TMR was calculated based on the DM content of each dietary ingredient. Daily TMR offer in each group was estimated to maintain about 3%, 5%, and 2% refusal in fresh, high, and late lactation cow groups, respectively. The DMI intake and refusal were adjusted weekly.

Two milk tests were carried out in each dairy unit to evaluate the milk production and composition. At time 0 (initial), and after 4 weeks of mix-tannins supplementation (final), all lactating cows in the two dairy units were evaluated for milk yield and milk composition to determine the effects of ByPro^®^. For the statistical analysis, cows were categorized according to days in milk (DIM) as follows, from: 0 to 30, 31 to 120, 121 to 365, and more than 365 DIM. Milk production per cow was measured with a DeLaval ALPRO equipment in each dairy unit. The initial and final milk samples were collected in 50 ml containers with 4 drops of Azidiol as milk preservative. The milk components were analysed with an equipment BENTLEY model FTS 500 (BENTLEY International Inc.) to measure: fat, protein, lactose, and total solids (TS) contents.

To characterize TMR nutrient contents, at the beginning and at the end of the milk production and composition evaluations, two samples of TMR were taken from the whole feed bank a the high production cows’ corrals in both dairy units (*n* = 2 per dairy unit) and analyzed for CP, ADF, NDF, Fat, Ash, Ca, P, Mg, Cl, K, Na, Cu, Fe, Mn, and Zn, by standard methodologies (AOAC 1990, 1999), as reported in previous studies (Cavallini et al. 2018, 2022).

The observation units were the milking cows, and the variables analyzed were milk yield/cow per day, fat corrected milk 4%/cow per day (FCM4%), milk fat, protein, lactose and total solids contents. The initial milk performance assay was to evaluate similarity between dairies with no treatment. The final data was used to evaluate the effect of ByPro^®^. A lineal model was adjusted for each variable. The model included the following treatment factors: dairy units (Control and ByPro) zmilk tests (initial and final), days in milk (DIM), lactation numbers (1 to 6), and their interactions. Due to animals being managed in different corrals and each animal were evaluated two times (initial and final milk tests), two random effects were included in the model: the corral and animals within the corral. The treatment effect (ByPro vs. Control) on each variable was estimated as a contrast between initial and final adjusted means: Contrast = ByPro (final – initial) – Control (final – initial). Models were adjusted using the *lme* function of the *nlme* package (Pinheiro et al. 2015) of the “R language” (R Core Team, 2015) in the interface of *InfoStat statistical software* (Di Rienzo, J.A, et al., 2015). To compare the effect of mix tannins on more homogeneous groups and to reduce variability, all animals with less than 10 L of milk/d and more than 70 L of milk were eliminated for the statistical analysis. Most of the cows with less than 10 L milk/d were with more than 365 DIM, for this reason all the cows in this category (> 365DIM) were not included in the statistical analysis.

## Results and discussion

This section explores the effects of a tannin-based feed additive on the productivity and composition of milk in lactating dairy cows. The analysis builds upon the premise that condensed and hydrolysable tannins may enhance nutrient utilization efficiency and support milk production under commercial dairy conditions. By comparing treated and control groups, the discussion contextualizes the findings within the broader framework of ruminant nutrition and evaluates their implications for practical dairy management.

Because of the restrictions of the assay, ByPro and Control effects may be confounded with dairy units. This drawback of on-farm research was taken into account on the modeling approach. Verifying that all relevant variables at the beginning of the assay, before any treatment was applied, were not different between dairy units. Likewise, the treatments effects were evaluated as the change in mean response between the end and beginning of the assay, or final and initial milk tests.

Estimated DMI and milk yield per cow were similar in both dairy farms averaging 25.5 kg DMI/cow and more 32 kg milk/cow per day (Table 1). Production values and the daily time budget were maintained within the normal physiological and behavioral parameters for lactating cows in this system (Lamanna et al. 2025b). Feed ingredients and nutrient contents in both dairies were also comparable (Table 1). The main difference between lactating dairy cows’ diet was the dietary supplied in the TMR of a mix of condensable and hydrolysable tannins feed additive ByPro^®^.

**Table 1:** TMR ingredients composition, estimated dry matter intake (DMI), milk yield/cow, and dietary nutrient content

item	ByPro Dairy	Control Dairy	
TMR ingredients ^**(1)**^			
Alfalfa hay, %			7.0
Corn silage, %	26.4		
Wheat silage, %	5.0		
Alfalfa silage, %	4.0		
Corn grain, high moisture, %	5.8		
Corn grain, ground dry, %	14.2		
Corn gluten feed, %	20.0		
Soybean meal, %	7.2		
Soybean grain, %	5.8		
Fat, bypass, %	1.1		
Premix, **%**	3.5		
DMI, kg/cow/day ^**(2)**^	25.70	25.40	
Milk yield, kg/cow/day ^**(2)**^	32.73	33.99	
Nutrient contents ^**(3)**^			
CP, %	17.40	17.75	
NDF, %	27.81	27.20	
ADF, %	19.10	18.00	
Fat, %	5.10	6.10	
Ash, %	8.84	8.39	
Ca, %	0.99	0.82	
P, %	0.42	0.47	
Mg, %	0.37	0.33	
K, %	1.43	1.35	
Na, %	0.20	0.19	
Cl, %	0.31	0.28	
Cu, ppm	16.50	14.50	
Mn, ppm	53.25	44.92	
Zn, ppm	62.42	57.25	

^1^ TMR ByPro Dairy includes the mix of tannins in the Premix

^2^ Averages at the initial of the experiment including all the lactating cows

^3^ Nutrient contents, *n* = 2, TMR samples were taken at the beginning (initial) and at the end (final) of milk production evaluations from the high milk production cows’ corral in each dairy

Tannin extracts are often associated with reduced DMI in ruminants due to decreased palatability and slower digestion rates (Kumar and Vaithiyanathan 1990; Aguerre et al. 2016). These adverse effects are typically linked to the type of tannins used or high dietary inclusion rates (≥ 3% of dietary dry matter) (Dschaak et al. 2011; Henke et al. 2017). For instance, Grainger et al. (2009) reported a reduction in DMI when condensed tannin extracts were included at levels as low as 0.9% of dietary DM. However, most studies have shown that tannin inclusion rates below 3% of dietary DM do not negatively impact DMI (Beauchemin et al. 2007; Liu et al. 2013; Henke et al. 2017), aligning with the findings of this study. Here, DMI remained unaffected, likely due to the low inclusion rate of tannins (0.3% of dietary DM). Furthermore, the results of Wang et al. (2022) suggest that supplementing early lactating cows with up to 30 g/d of tannins is a practical and feasible approach under similar conditions.

Table 2 shows the number of cows evaluated in this experiment and the proportion of cows in each category based on DIM. The lowest proportions of cows were in the category 0–30 (about 10%) and cows with more than 365 DIM (8%). Cows from 30 to 120 and from 121 to 365 DIM represented more than 80% of the total cows. More than 5400 samples from individual lactating dairy cows were analyzed in ByPro (milk yield per cow and milk chemical analysis), including both, initial and final milk test; and 6422 samples in the Control.

**Table 2:** Number of lactating cows in each dairy categorized according to days in milk (DIM)

Dairy/treatment	DIM	Cows evaluated ^(1)^	Proportion/category
ByPro^®^	0 to 30	263	0.11
ByPro^®^	31 to 120	785	0.26
ByPro^®^	121 to 365	1462	0.55
ByPro^®^	More than 365	208	0.08
*Total*		2717	1.00
Control	0 to 30	348	0.10
Control	31 to 120	829	0.28
Control	121 to 365	1752	0.54
Control	More than 365	284	0.08
*Total*		3211	1.00

^1^ Each cow was evaluated two times (initial and final milk test), cows with more than 365 DIM were not included in the statistical analysis

Results of milk production and composition including all lactating animals are presented in Table 3. A significant increment (*P <* 0.01) of milk yield per cow (+ 2.3 ± 0.33 L milk/cow per d) was obtained with no effect on milk fat and protein contents. Lactose content was reduced in both dairies, less in Control respect to ByPro. Because of milk improvement, productivity of milk fat and protein (kg/d) were higher in ByPro (Table 3).

**Table 3:** Milk production and composition including all lactating cows in each dairy farm (ByPro^®^ and Control)

		Dairy ByPro^®^		Dairy Control				
		initial	final	initial	final	Contrast ^(1)^	SEC ^(**2)**^	*P*-values ^(3)^
Milk yield	kg/d	32.73	33.89	33.99	32.81	2.34	0.331	**
FCM4% ^(**4)**^	kg/d	32.67	33.04	34.29	32.01	2.64	0.424	**
Fat	%	4.01	3.99	4.01	4.02	-0.03	0.048	ns
Protein	%	3.18	3.28	3.33	3.41	0.02	0.024	ns
Lactose	%	5.21	5.03	5.17	5.02	-0.03	0.014	*
Total solids	%	13.17	13.09	13.34	13.23	0.02	0.061	ns
Fat	kg/d	1.30	1.30	1.38	1.27	0.11	0.022	**
Protein	kg/d	1.03	1.08	1.14	1.07	0.12	0.013	**
Lactose	kg/d	1.72	1.71	1.77	1.65	0.11	0.018	**
Total solids	kg/d	4.30	4.35	4.55	4.25	0.37	0.048	**

^1^ Contrast = ByPro (final – initial) – Control (final – initial)

^2^ SEC = standard errors of the contrasts

^3^ *P*-values: ** = *P* < 0.01; * = *P* < 0.05; and **ns** = non-significant

^4^ FCM4% = fat corrected milk 4%

Milk yield in early lactation cows (0–30 DIM) was not different between ByPro^®^ and Control; while fat, protein and total solids were higher in the Control (Table 4). The Standard Errors of the Contrasts (SEC) for fat, protein, and lactose milk contents in cows between 0 and 30 DIM were high (Table 4) more than 2.2 times when compared to all animals (Table 3). The high concentrations of milk fat and protein at the final milk test (e.g. Table 4; more than 4.40% of fat and more than 3.50% protein, in both ByPro and Control) might be related to a higher number of cows close to calving date. This reason and the high variability obtained during this period (0–30 DIM) makes it difficult to compare both dairy units.

**Table 4:** Milk production and composition in early lactation dairy cows (0–30 DIM)

		Dairy ByPro^®^		Dairy Control				
		initial	final	initial	final	Contrast ^(1)^	SEC ^(2)^	*P*-values ^(3)^
Milk yield	kg/d	31.03	33.59	32.85	34.17	1.25	0.807	ns
FCM4% ^(**4)**^	kg/d	33.29	35.04	32.59	33.75	0.59	0.999	ns
Fat	%	4.42	4.31	4.06	4.47	-0.56	0.110	**
Protein	%	3.28	3.23	3.35	3.50	-0.21	0.054	**
Lactose	%	5.10	5.01	5.13	4.91	0.14	0.032	**
Total solids	%	13.57	13.33	13.39	13.63	-0.48	0.137	**
Fat	kg/d	1.38	1.44	1.29	1.37	-0.02	0.050	ns
Protein	kg/d	1.02	1.07	1.11	1.10	0.06	0.030	*
Lactose	kg/d	1.60	1.69	1.70	1.67	0.12	0.077	**
Total solids	kg/d	4.24	4.47	4.37	4.43	0.18	0.115	ns

^1^ Contrast = ByPro (final – initial) – Control (final – initial)

^2^ SEC = standard errors of the contrasts

^3^ *P*-values: ** = *P* < 0.01; * = *P* < 0.05; and **ns** = non-significant

^4^ FCM4% = fat corrected milk 4%

The main responses of cows supplemented with a mix of tannins ByPro^®^ were between 30 and 356 DIM (Tables 5 and 6). Mid-lactation cows among 31–120 DIM increased (*P <* 0.01) milk yield per cow + 3.6 ± 0.42 L milk/d; and late lactation cows (121 to 365 DIM) + 3.2 ± 0.44 L milk/d. Significant responses in ByPro^®^ dairy unit on milk fat and protein content from 31 to 120 and 121–365 DIM were observed: +0.27 ± 0.065% fat, + 0.21 ± 0.032% protein (*P* < 0.01), and + 0.17 ± 0.069% fat, + 0.11 ± 0.034% protein (*P* < 0.05), respectively.

**Table 5:** Milk production and composition in lactating dairy cows from 31–120 DIM

		Dairy ByPro^®^		Dairy Control				
		initial	final	initial	final	Contrast ^(1)^	SEC ^(2)^	*P*-values ^(3)^
Milk yield	kg/d	36.63	39.29	37.54	36.58	3.62	0.420	**
FCM4% ^(**4)**^	kg/d	35.57	37.45	38.21	35.36	4.73	0.553	**
Fat	%	3.72	3.78	3.96	3.75	0.27	0.065	**
Protein	%	2.97	3.04	3.29	3.16	0.21	0.032	**
Lactose	%	5.28	5.08	5.18	4.07	-0.09	0.018	**
Total solids	%	9.00	8.94	9.30	9.05	0.19	0.036	**
Fat	kg/d	1.38	1.45	1.53	1.37	0.22	0.029	**
Protein	kg/d	1.10	1.19	1.27	1.15	0.21	0.016	**
Lactose	kg/d	1.95	2.01	1.96	1.87	0.16	0.024	**
Total solids	kg/d	4.70	4.96	5.06	4.68	0.64	0.062	**

^1^ Contrast = ByPro (final – initial) – Control (final – initial)

^2^ SEC = standard errors of the contrasts

^3^ *P*-values: ** = *P* < 0.01; * = *P* < 0.05; and **ns** = non-significant

^4^ FCM4% = fat corrected milk 4%

**Table 6:** Milk production and composition in lactating dairy cows from 121 to 365 DIM

		Dairy ByPro^®^		Dairy Control				
		initial	final	initial	final	Contrast ^(1)^	SEC ^(2)^	*P*-values ^(3)^
Milk yield	kg/d	34.23	35.43	35.50	33.48	3.22	0.442	**
FCM4% ^(**4)**^	kg/d	33.15	33.64	35.67	32.21	3.96	0.588	**
Fat	%	3.82	3.80	3.96	3.77	0.17	0.069	*
Protein	%	3.14	3.30	3.30	3.34	0.11	0.034	**
Lactose	%	5.29	5.06	5.19	5.06	-0.10	0.020	**
Total solids	%	13.02	12.96	13.27	12.97	0.23	0.085	ns
Fat	kg/d	1.30	1.30	1.43	1.26	0.18	0.031	**
Protein	kg/d	1.07	1.14	1.18	1.09	0.15	0.017	**
Lactose	kg/d	1.81	1.79	1.85	1.70	0.13	0.025	**
Total solids	kg/d	4.44	4.52	4.73	4.31	0.51	0.065	**

^1^ Contrast = ByPro (final – initial) – Control (final – initial)

^2^ SEC = standard errors of the contrasts

^3^ *P*-values: ** = *P* < 0.01; * = *P* < 0.05; and **ns** = non-significant

^4^ FCM4% = fat corrected milk 4%

Milk yield and milk composition (contents and production) improved on more than 80% of the cows receiving the mix of tannins. Based on previous studies (Aguerre et al. 2015, 2016) these results might be related to changes and improvements on nutrient utilization efficiency. Different studies indicate that tannins may play an important role in improving energy utilization efficiency in ruminants. For example, microorganism resident in the gut of multicellular organisms, termed gut microbiome, have been shown to play important functions in their host’s physiology (Arumugam et al. 2011). This relationship was described with potential in the bovine rumen microbiome modulating feed efficiency and milk production performance (Jami et al. 2014). Studies of Min et al. (2014) demonstrated that rumen microbiota population can be changed when grazing goats were supplemented with chestnut and quebracho tannin extracts affecting VFA rumen production and modulating microbiota contents of Bacteroides and Firmicutes phylum. Other studies analyzed by Goel and Makkar (2012), discussed different mode of actions of tannins to reduce rumen methanogenic microorganisms, by lowering feed degradation rates and/or competing for hydrogen with the methane synthesis, and increasing efficiency of dietary energy utilization. Martinez et al. (2006), using tannic acid and quebracho tannins demonstrated how tannins affected microbial rumen fermentation of starch rich concentrates. Tannins did not prevent rumen bacterial attachment to starch granules, but starch hydrolysis was slowed indirectly because of a tannin mediated reduction in the degradation of the surrounding protein matrix, which should increase the energy-starch utilization efficiency.

The ability of tannins to bind proteins have been well described. Tannins that bind to dietary protein increase the N flux from the rumen to the small intestine. This process has been referred to as “ruminal escape protein” (Muller-Harvey 2006). In fact, the observed positive effects on milk yield and milk protein yield may be partly explained by an increased supply of rumen-undegradable protein (RUP), facilitated by the formation of tannin-protein complexes. In-vitro studies with *Leucana leucocephala* tannins lead to the hypothesis that tannins-protein complexes are formed at the pH rumen prevalent (pH 6–7) and that post-ruminal pH shifts in the abomasum (pH < 3.5) and the small intestine (pH > 7) release protein from these complexes, thus making it available for gastric and pancreatic digestion (quoted by Muller-Harvey 2006). The same author analyzes other tannins, reaching the conclusion that not all tannins behave in the same way. The extent to which protein digestion is affected by tannins depends on the source of tannins and the tannins concentration in the diet.

A mix of tannins research with high production performance lactating dairy cows was carried-out at Wisconsin University (Aguerre et al. 2015, 2016). Results are very consistent in both studies. Feeding a mix tannin of quebracho and chestnut tannin extract at 0.45% of diet DM may be adequate to reduce excretion of environmental labile urinary N and increase N utilization efficiency by improving true protein content in milk, and reducing milk urea-N and N-excretion. The dietary CP level in both experiments was estimated with NRC (2001), RDP and RUP levels very close to animal requirements. Animals were very similar (low variability) in terms of production performance, averaging about 40 and almost 50 kg milk/cow per d in 2015 and 2016 studies, respectively. Comparing to the Wisconsin results, where no effect on milk yield per cow was observed even on very high levels of tannins in the diets, in this on-farm research with a lower level of mix tannins in the diet, responses in milk yield were high, and important differences on milk fat and protein contents were obtained. These differences might be related to the high variability in milk yield per cow and higher dietary (over animal requirements, see Table 1) CP levels, which are used in commercial dairies farms to cover production variability of cows in the same corrals with different milk production and requirements. The mix tannins might help to balance the unbalance diets (excess of protein) in corrals where different levels of milk production cows are together with same diets. Protein balances in diets with high levels of dietary CP and RDP on low production cows might be improved increasing “ruminal escape protein” reducing rumen ammonia hepatic-way to produce urea and increasing RUP. The estimated CP balance (Table 1), give an average positive balance of RDP + 120 g/d, RUP + 470 g/d, and MP + 390 g/d, which support the hypothesis that low production cows were overfed dietary protein.

Aguerre et al. (2016), concluded that increasing levels of a tannins extract mixture from quebracho and chestnut in the diet had negative effects on DMI, fat-protein corrected milk, milk true protein content, and nutrient apparent digestibility, when fed at 0.90% and 1.80% of diet DM. Nevertheless, positive results were obtained with 0.45% of diet DM with quadratic responses in both experiments indicating that lower doses should be evaluated. This conclusion, and our results with lower level of mix tannins (0.3% diet DM) indicate that more research needs to be done to find the more efficient dose for lactating dairy cows combining different types of tannins among 0.0 and 0.45% of the dietary DM.

This study has some minor limitations. First, the research was conducted under specific on-farm conditions, which may limit the generalizability of the findings to other management systems or geographic regions. Second, while the study assessed overall lactation performance and nitrogen utilization, it did not evaluate detailed rumen microbial changes or antioxidant status, which could provide deeper insights into the mechanisms underlying the observed effects. Third, the study’s short duration may not capture the long-term impacts of tannin supplementation on animal health, productivity, or environmental outcomes. Lastly, potential confounding effects, such as variations in individual cow responses or management practices between experimental groups, could not be fully eliminated due to the inherent constraints of on-farm research designs.

Future research should explore the mechanisms behind tannin supplementation, including its effects on rumen microbiota, fermentation, and antioxidant status, while assessing its long-term impacts on health, productivity, and sustainability.

## Conclusions

This large-scale, on-farm study demonstrates that supplementing dairy cows with a commercial tannin mix can significantly improve production performance. Cows between 30 and 365 DIM represented more than 80% of the total cows evaluated in this research. Supplementation with the tannin mix ByPro^®^ increased lactation performance compared with the control group. When considering all lactating cows included in this study (0–365 DIM), milk yield increased by + 2.3 L/cow per day, with no effects on milk fat or protein content. The most notable effects were observed in mid- and late-lactation cows. Mid-lactation cows (31–120 DIM) showed increases in milk yield (+ 3.6 L/cow per day), milk fat (+ 0.27%), and milk protein (+ 0.21%). Late-lactation cows (121–365 DIM) showed increases in milk yield (+ 3.2 L/cow per day), milk fat (+ 0.17%), and milk protein (+ 0.11%).These findings suggest that tannins can be a valuable tool for dairy producers to enhance profitability and improve nitrogen utilization under typical commercial management conditions.

## Data Availability

The data for this study are available from the corresponding author upon reasonable request.
